# Health state utilities associated with X-linked retinitis pigmentosa (XLRP)

**DOI:** 10.1007/s10198-025-01761-y

**Published:** 2025-03-17

**Authors:** Louis S. Matza, Nan Li, Katie D. Stewart, Mahmoud Hashim, Tom Denee, Feng Pan, Qiaoyi Zhang, Jennifer Lee, Michel Michaelides, Hendrik P. N. Scholl

**Affiliations:** 1 Evidera, 929 North Front Street, Wilmington, NC 28401-3331 USA; 2 Janssen Pharmaceutical K.K, Tokyo, Japan; 3 Janssen Global Services, LLC, Beerse, Belgium; 4 Janssen Europe, Middle East and Africa, Breda, The Netherlands; 5 Janssen Global Services, LLC, Raritan, NJ USA; 6 Janssen Europe, Middle East and Africa, Copenhagen, Denmark; 7 UCL Institute of Ophthalmology and Moorfields Eye Hospital, London, UK; 8 Department of Clinical Pharmacology, Medical University of Vienna, Vienna, Austria; 9 Pallas Kliniken AG, Pallas Klinik Zürich, Zürich, Switzerland; 10 European Vision Institute, Basel, Switzerland

**Keywords:** Health state utility, X-linked retinitis pigmentosa, XLRP, Visual impairment, Rare disease, Time trade-off

## Abstract

**Background:**

X-linked retinitis pigmentosa (XLRP) is a rare, inherited retinal disease characterized by impairment in visual field and visual acuity with continuous progression leading to blindness. Gene therapies for XLRP are under investigation, and health state utilities are needed for use in cost-utility analyses examining the value of these treatments.

**Objective:**

This study aimed to estimate utilities associated with XLRP severity.

**Methods:**

Eleven health state vignettes depicting combinations of impairment in visual field and visual acuity associated with XLRP were developed based on literature review and input from clinicians, patients, and a caregiver. Vignettes included text describing visual acuity impairment, visual field impairment, night blindness, impact on quality of life, and two images representing the combination of visual field and visual acuity impairment for each health state. Health states were valued in time trade-off interviews with general population respondents in the UK.

**Results:**

A total of 245 participants completed interviews (51.0% female; mean age = 41.4 years; Newcastle, *n* = 80; London, *n* = 85; Edinburgh, *n* = 80). In a ranking task, participants preferred health states with less severe visual impairment, and this preference was reflected in the utilities. Mean (standard deviation) utilities ranged from 0.900 (0.121) for the health state with no visual acuity impairment and mild visual field impairment to 0.271 (0.478) for the health state describing blindness.

**Conclusion:**

Results highlight the substantial impact of visual impairment on health state preference and quality of life. The health state utilities estimated in this study may be appropriate for use in cost-effectiveness models evaluating treatments for XLRP.

**JEL CLASSIFICATION CODES:**

I1; I12; I19

**Supplementary Information:**

The online version contains supplementary material available at 10.1007/s10198-025-01761-y.

## Introduction

Retinitis pigmentosa (RP) is a group of rare inherited retinal diseases (IRDs) characterized by night blindness and progressive impairment in visual field and acuity, gradually leading to blindness [1–7]. The most severe form of RP is X-linked RP (XLRP), characterized by early onset and continuous disease progression over many years [8, 9]. Because the condition is X-linked, it most commonly affects men, with symptoms typically starting by age 10 and progressing to legal blindness by a median age of 45 years [1, 8, 10].

Although there are no currently available therapies for XLRP, gene therapies have shown potential to improve retinal sensitivity [11–14]. As new treatments for XLRP are developed and proposed for use in various countries, cost-utility analyses (CUAs) will be needed to examine their value. For calculation of quality-adjusted life years, these CUAs will require utilities representing preference for health states associated with various severity levels of XLRP.

No previous studies reporting XLRP utilities were located. Two studies have reported utilities for other IRDs, but both have limitations. In one of these studies [15], neither patients nor general population respondents valued the health state vignettes. Instead, a small group of clinicians (*N* = 6) completed the EQ-5D and the Health Utilities Index Mark 3 (HUI3) based on their perceptions of hypothetical patients described in vignettes. This clinician-based approach is not a widely accepted method for estimating utilities. The second study estimated utilities of five health states describing RP (i.e., not specifically XLRP) based on preferences of a general population sample (*N* = 110) [16]. There are several limitations associated with these five health states. First, no utilities are provided for mild RP health states. Second, the limited number of health states does not allow for various combinations of visual acuity and visual field that commonly occur with XLRP, where impairment in visual field generally precedes impairment in visual acuity. Third, these health states described a specific emotional reaction to the visual impairment, which is not necessarily accurate for patients with XLRP. For example, clinicians and patients interviewed as part of the current study reported that XLRP symptoms are not necessarily associated with negative emotional reactions (see Health State Development section below). Given the limitations of these two studies and the fact that neither focused on XLRP, it appears that there are no published utility values that would be suitable for use in CUAs of treatments for XLRP.

Therefore, this vignette-based study aimed to estimate utilities associated with various severity levels of XLRP. Vignette-based methods were selected to overcome inadequate sensitivity of generic preference-based instruments in visual conditions [17–22], as well as the infeasibility of gathering data from sufficient numbers of patients with a rare disease. Following recent recommendations [23, 24], health state vignettes were based on published literature as well as input from patients, clinicians, and a caregiver.

## Methods

### Justification of study design

Several health technology assessment (HTA) guidelines recommend that CUAs use utilities derived from patient-completed generic preference-based measures (GPBMs), such as the EQ-5D, when these measures are feasible and appropriate [25–27]. Prior to designing the current study, GPBMs were considered for assessment of patients with XLRP.

The EQ-5D does not specifically assess the impact of visual functioning, and previous studies have highlighted the limited sensitivity of the EQ-5D in populations with visual impairment. EQ-5D scores often have low correlations with clinical measures of visual function and fail to differentiate between levels of clinical severity [17, 18, 20, 22]. In several studies, the EQ-5D has been unable to differentiate between people with and without visual impairment [19, 21]. The HUI3 may be more relevant in some visual conditions because it includes a vision dimension. However, this dimension focuses on visual acuity rather than visual field, which is the key differentiator among XLRP severity levels (definitions of visual acuity and visual field are presented in the Health State Descriptions section below) [8].

Condition-specific utility measures were also considered. The VFQ-UI focuses on quality of life associated with vision [28], but its six items do not appear to be directly relevant to the key features of XLRP. For example, the VFQ-UI dimensions do not assess impact of impaired visual acuity or reduced visual field. Furthermore, data suggest that this infrequently used instrument may have limited sensitivity to disease severity [21]. Another possibility is the “bolt-on” version of the EQ-5D with a sixth dimension assessing vision [29]. However, the vision dimension is global without any specificity in the type of visual impairment or the impact of vision loss, and the three response options (no problems, some problems, or extreme problems seeing) do not allow for much sensitivity to change. In sum, the available generic and condition-specific utility measures are unlikely to be useful for distinguishing among XLRP severity levels.

Another challenge with estimating utilities for XLRP is that RP is a rare disease, and only about 6% of RP is X-linked [1]. Therefore, it would be very difficult to recruit enough patients to provide reliable utility estimates for all relevant disease severity health states needed to support economic modeling.

Due to these measurement and feasibility issues with patient-based approaches, utilities associated with XLRP were estimated in the current study using vignette-based methods. Vignette-based utility studies are often used to estimate utilities for rare diseases when it is not feasible to collect preference-based data from a large enough sample of patients [23]. This approach is suggested in the National Institute for Health and Care Excellence guidelines as an alternative to consider when generic instruments are not feasible to administer or sensitive to the relevant medical condition [24, 26].

### Health state development

#### Health state descriptions

Health states were developed to represent various severity levels of XLRP. Health state severity was categorized according to visual acuity and visual field. Visual acuity is how clear and sharp objects appear [30]. Visual field is how wide of an area the eye can see [31]. Health state development was based upon published literature [30–33], design of a phase 1/2 XLRP clinical trial (NCT03252847) and a natural history study (NCT03349242), and interviews with clinicians, patients with XLRP, and a caregiver of an adult with XLRP.

Four clinicians (from Switzerland, Netherlands, US, UK) were consulted throughout health state development to ensure that the health states were consistent with clinical impressions. Each of these advisors had at least 10 years of experience working with patients with XLRP. Each clinician was involved in multiple meetings to discuss the health states, and they were also consulted via email. In initial interviews, they suggested combinations of severity in visual acuity and visual field impairment to represent the patients most typically seen in clinical practice. Definitions of severity of visual acuity and visual field were based on the International Council of Ophthalmology visual standards report [33], as summarized in Online Resource 1. Based on these sources of information, 14 combinations of visual acuity and visual field impairment were considered to represent common profiles of XLRP and were therefore selected to be health state vignettes. During the pilot study, the number of health states was reduced from 14 to 11, as described below.

Clinicians were interviewed further to inform development and refinement of the health state content, while ensuring that the descriptions were clear and accurate representations of typical patient experiences with XLRP. Interviews focused on eliciting descriptions of various levels of impairment in visual acuity and visual field, as well as descriptions of other visual symptoms (e.g., night blindness), impact on activities, and impact on quality of life. Clinicians considered night blindness to be an important feature of XLRP, and they provided descriptions of four levels of night blindness for use in the health states. Areas of impact described by clinicians included self-care, daily activities, emotions, and driving. Clinicians were asked to describe these topics in terms that would be comprehensible for general population respondents and would clearly distinguish between levels of impairment.

According to clinicians, the emotional impact of XLRP varies more by individual than by severity of impairment. They said most patients experience concerns about the future, but the level of concern or anxiety is typically related to the patient’s personality more than their severity of visual impairment. Therefore, a single general statement about emotional impact was used in all health states to highlight potential concern for the future and impact on emotions, without providing specific assumptions about emotional status.

Clinicians reported that most patients with XLRP do not drive, but some patients with mild-to-moderate impairment drive during the day. Legal requirements for driving vary geographically, and a statement was included in the health states to acknowledge that driving would not be legally permitted in some locations.

A brief description of XLRP was developed for a background information page to be presented to respondents prior to the health states. This description was based on guidelines from relevant associations [30–32] and input from clinicians. The background information page also provided brief non-technical definitions of visual acuity (“how clear and sharp objects appear”) and visual field (“how wide of an area your eye can see”) to ensure that participants understood the key concepts and terminology included in the health states. All materials omitted the condition’s name to avoid potential biasing that may be associated with labeling [34]. Instead, XLRP was introduced as a “rare genetic eye disease that causes blindness over time.” The final versions of the background information page and health state vignettes are presented in Online Resource 2 and Online Resource 3, respectively. All clinicians approved these documents prior to their use in the utility elicitation study.

#### Images for health states

**Fig. 1:**
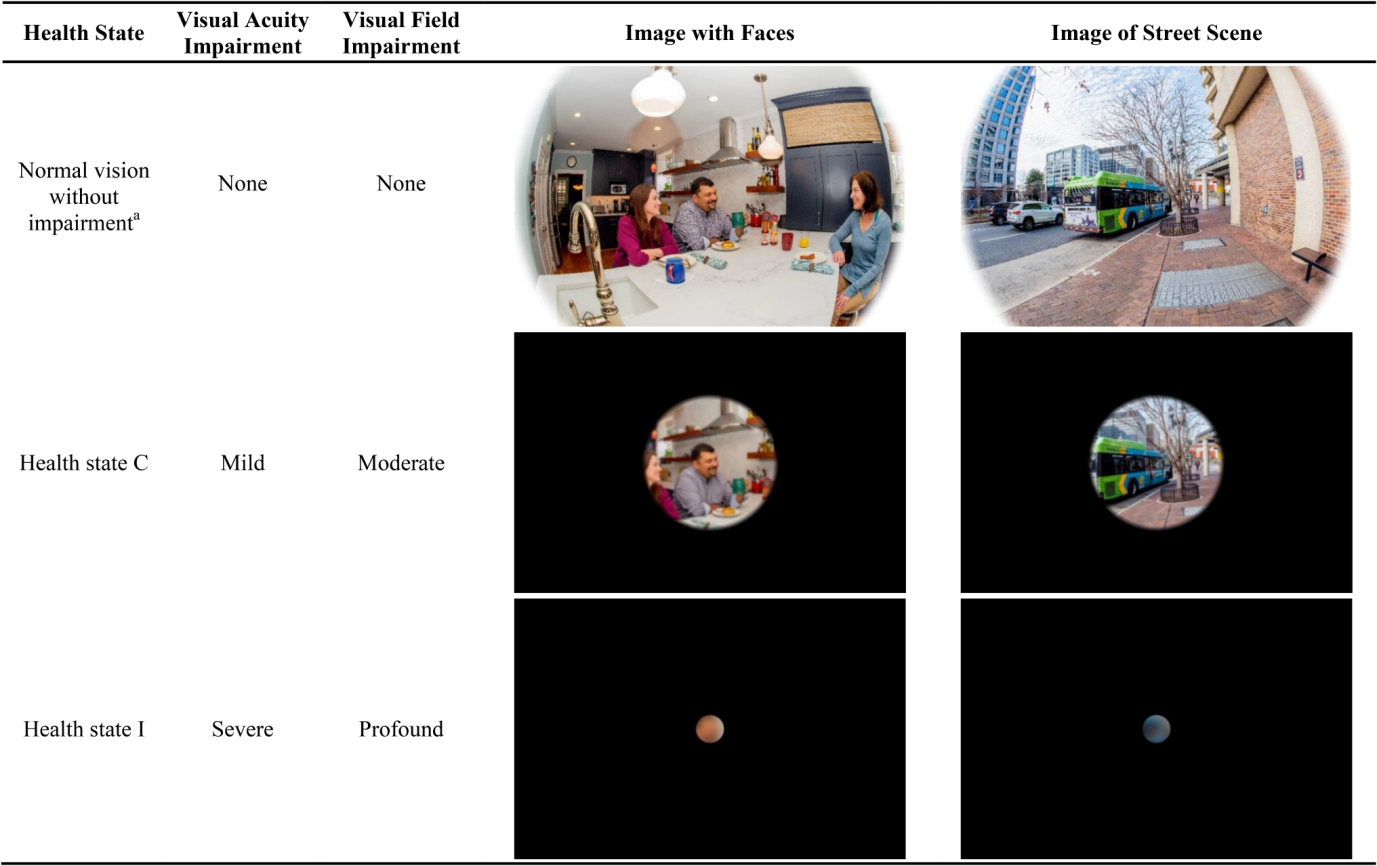
Examples of Images Used in Health States. ^a^ Images of normal vision without impairment were shown to the participants with the background information page to provide context for the health states representing visual impairment

Two images were included in each health state to illustrate the level of visual impairment described in the text, one image of a street scene to highlight visual field and one image with faces to highlight visual acuity. A professional photographer was hired to take photographs using a wide-angle lens to best simulate the full visual field for a person with normal vision (see images in Fig. 1). In the utility elicitation interviews, the full images were presented to provide an indication of normal vision.

For each health state, the images were altered to represent the specific levels of impairment in visual field and visual acuity. To simulate levels of visual field impairment, images were altered by restricting the diameter of the image around a central point. The diameter used for each level of visual field impairment was based on the upper limit of the visual field range as presented in Online Resource 1, except for the “blind” health state, which was represented with a black box. Sight-Sim was used to simulate levels of visual acuity loss using the LogMar values presented in Online Resource 1. Sight-Sim is a Java-based program that can simulate visual impairment based on visual acuity. Gaussian blur was applied to the photographs in Adobe Photoshop to match the Sight-Sim simulation. The clinicians approved the final images in all health states.

#### Patient and caregiver validation of health states

Interviews were conducted with three men with XLRP and a carer (wife) of one of these patients. Each patient and caregiver was asked to review and comment on the background description of XLRP, the health state most closely matching their current condition, and any health states corresponding to their previous experiences with XLRP. All three patients and the carer agreed that the health state content was accurate with regard to visual field and visual acuity impairment. Patients agreed with clinicians that the emotional impact of XLRP varies widely, but concern for the future was common.

However, they also agreed that the text initially drafted using clinician input underestimated their functional abilities and levels of independence. For example, health state language drafted with clinicians included a statement for all health states with profound or greater impairment in visual acuity indicating that “you are unable to perform daily activities without assistance.” In contrast, patients and the caregiver reported that they are able to accomplish many daily activities independently such as making the bed, doing laundry, folding clothes, getting to familiar places with public transportation, and preparing some foods. Patients reported needing assistance with other activities such as finding items around the house, managing finances, shopping, and getting around unfamiliar places. Patient and caregiver feedback was reviewed with the clinicians. The clinicians recommended prioritizing the patient feedback and revising the health states to reflect patient experiences with daily activities more accurately. The revised health states were then reviewed by a patient and the caregiver, who both approved the revisions prior to the pilot study.

### Pilot study

A pilot study was conducted in London, UK (*N* = 20 general population participants; mean age 52.8 years; 55.0% male). Participants completed the time trade-off (TTO) utility elicitation task and provided feedback on the health states and interview procedures. The pilot study began with 14 health states. Some participants had difficulty ranking the health states due to the volume of information. The number of health states was gradually reduced until participants could consistently complete the interviews more easily, resulting in a final set of 11 health states for the main valuation study. All participants reported that the health states and background information page were clear and comprehensible.

Minor revisions to health state wording were made after receiving feedback from pilot participants. For example, the term “profound” was revised to the more easily comprehensible “very severe.” The minor revisions to the health states were discussed with clinicians to ensure the final health states were accurate and reasonable.

### Participants

The current study was conducted with general population respondents for consistency with HTA preferences for the general population perspective in utility elicitation [26]. Digital social media marketing (e.g., Facebook, Twitter, Google) was used to recruit participants. Interested participants responded to advertisements and were screened by telephone for eligibility. Participants were required to be over 18, a resident of the UK, able and willing to give informed consent, and able to complete the protocol requirements. Respondent sex, age, racial/ethnic background, and employment status were tracked during recruitment in an effort to recruit a sample reflective of the UK’s general population with regard to these variables. For the safety of interviewers and participants, all study personnel and participants were required to be vaccinated against COVID-19.

### Utility interview procedures and scoring

Utilities for the 11 health states were elicited in one-on-one, in-person TTO interviews in May 2022 in three UK cities (Newcastle, London, Edinburgh). Interviews were conducted by nine trained interviewers (supervised by the principal investigator and project manager) according to a semi-structured interview guide. Participants provided informed consent, and the study was approved by an independent institutional review board (Ethical & Independent Review Services; Study Number 22020-01).

Participants were first shown the background information page describing XLRP and listing seven levels of impairment in visual acuity and visual field (Online Resource 2). The background information page was accompanied by two unaltered images representing “normal vision” to provide context for the altered images that appear with each health state (see Fig. 1 for “normal vision” images). Participants were then introduced to the health state descriptions presented on individual cards in random order, and they completed a ranking exercise (from most preferable to least preferable) to help familiarize them with the health state content and consider their preferences before the TTO task.

After completing the ranking, participants valued the health states in a TTO task with a 10-year time horizon. For each health state, participants were offered a choice between 10 years in the health state and varying amounts of time in full health. TTO choices were presented in 6-month (5%) increments, alternating between longer periods of time and shorter periods of time in full health (i.e., 10 years, 0 [dead], 9.5, 0.5, 9, 1…). Utility (*u*), with the anchors of dead (0) and full health (1), was assigned based on the point of indifference between *x* years in full health and *y* years in the health state and calculated as *u = x/y*.

Utilities for health states perceived to be worse than dead were elicited by adjusting the TTO task as described in previous literature [35]. When a participant said a health state was worse than dead, they were presented with a series of choices between dead and a 10-year life span beginning with varying amounts of time in the health state being rated followed by full health for the remainder of the 10-year period. The resulting negative utility was calculated as *u=-x/10*, where *x* is time in full health.

### Statistical analysis procedures

Statistical analyses were completed with SAS (version 9.4, SAS Institute, Cary, NC). Descriptive statistics were used to summarize demographic data, health state rankings, and utilities (means and standard deviations for continuous variables; frequencies and percentages for categorical variables). Paired *t* tests were conducted to test whether there were statistically significant pairwise differences between health state utilities (e.g., adjacent pairs of health states). Independent *t* tests were conducted to test for differences in utility by age, sex, and country (England and Scotland).

## Results

### Sample characteristics

A total of 275 participants were scheduled, and 251 attended interviews. Six of the 251 had difficulty understanding the utility interview procedures and were unable to provide valid data. Therefore, the analyses were conducted with a sample of 245 participants, including 165 in England (Newcastle, *n* = 80; London, *n* = 85) and 80 participants in Edinburgh, Scotland. Demographics are presented in Table 1. The most common health conditions were anxiety (25.3%), depression (24.1%), hypertension (8.6%), and arthritis (8.2%). No participants reported having been diagnosed with RP, but three (1.2%) reported knowing someone with RP.

**Table 1:** Demographic Characteristics

Characteristics	Descriptive Statistics (*N*=245)
**Age**, **Mean years (SD)**	41.4 (15.6)
**Gender**, **n (%)**	
Male	119 (48.6%)
Female	125 (51.0%)
Nonbinary	1 (0.4%)
**Ethnic/Racial background**, **n (%)**	
Asian/Asian British	18 (7.3%)
Black/African/Caribbean/Black British	5 (2.0%)
White	212 (86.5%)
Mixed/multiple ethnic groups	5 (2.0%)
Other	5 (2.0%)
**Marital status**, **n (%)**	
Single	99 (40.4%)
Married	74 (30.2%)
Other^a^	72 (29.4%)
**Employment status**, **n (%)**	
Full-time work	111 (45.3%)
Part-time work	48 (19.6%)
Other^b^	86 (35.1%)
**Education level**, **n (%)**	
University degree	140 (57.1%)
No university degree	105 (42.9%)
**Interview location**, **n (%)**	
Newcastle, England	80 (32.7%)
London, England	85 (34.7%)
Edinburgh, Scotland	80 (32.7%)

*Abbreviation*: SD = standard deviation

^a^ Other marital status includes divorced (*n*=21), separated (*n*=7), widowed (*n*=2), cohabitating/living with a partner (*n*=38), and not specified (*n*=4)

^b^ Other employment status includes homemaker (*n*=11), student (*n*=24), unemployed (*n*=12), retired (*n*=26), and not specified (*n*=13)

### Health state rankings and preferences

Health state rankings ranged from 1 (most preferable) to 11 (least preferable). Participants tended to prefer health states with less visual impairment over health states with more severe impairment (see Table 2 for levels of visual impairment in each health state). Health states A and B were ranked as the most and second most preferable by all participants. Health state K (blind) was the least preferred health state for nearly all participants (91.8%). Participants ranking blind as the least preferred health state mentioned needing assistance, being a “burden,” and missing visual experiences like color and seeing family. Quotations from participants ranking blind as least preferred include: “It’s a big affliction to me, like not being able to take in people’s faces or the environment around you or colour”; “I’m very visual, I love colour…it’s most important”; “You need a lot of help with everything and I don’t feel like you could enjoy things the same way”; “You would have no independence whatsoever, you couldn’t see any new members of the family.”

**Table 2:** Comparison of Health State Utilities between Male and Female Participants^a^

Health State			Health State Utility			T test	
Health State Label	Visual Acuity	Visual Field	Male (*N*=119) Mean (SD)	Female (*N*=125) Mean (SD)	Difference Mean (SD)	t statistic	*P* value
A	No impairment	Mild	0.881 (0.150)	0.916 (0.083)	-0.035 (0.120)	-2.3	0.026
B	No impairment	Moderate	0.841 (0.170)	0.888 (0.104)	-0.046 (0.140)	-2.6	0.011
C	Mild	Moderate	0.798 (0.191)	0.847 (0.137)	-0.050 (0.166)	-2.3	0.021
D	Mild	Severe	0.674 (0.285)	0.747 (0.199)	-0.073 (0.245)	-2.3	0.022
E	Mild	Profound	0.538 (0.366)	0.661 (0.237)	-0.123 (0.307)	-3.1	0.002
F	Moderate	Severe	0.566 (0.339)	0.680 (0.212)	-0.114 (0.281)	-3.1	0.002
G	Moderate	Profound	0.475 (0.376)	0.613 (0.251)	-0.138 (0.318)	-3.4	<0.001
H	Moderate	Near blind	0.324 (0.446)	0.499 (0.276)	-0.175 (0.369)	-3.7	<0.001
I	Severe	Profound	0.303 (0.466)	0.493 (0.293)	-0.189 (0.387)	-3.8	<0.001
J	Severe	Near blind	0.237 (0.492)	0.405 (0.370)	-0.168 (0.433)	-3.0	0.003
K	Blind	Blind	0.197 (0.496)	0.337 (0.450)	-0.141 (0.473)	-2.3	0.021

*Abbreviation*: SD = standard deviation

^a^ One participant reported their gender as nonbinary. This participant has been excluded from this analysis because of insufficient sample size for comparison to participants self-identifying as male or female

The 20 (8.2%) participants who preferred health state K (blind) over at least one other health state thought that being blind would be less “irritating,” “distracting,” “frustrating,” or “stressful” than having severely impaired vision. Example quotations from participants who did not rank blind as the least preferred health state include: “For [health states J and I], I would find it very stressful and upsetting to be able to see but not see. I would rather just be blind”; “If I had vision that was going to keep getting worse, I’d rather be blind than have the anxiety of worrying about what was coming”; “Being blind would be the worst, but these would be a lot scarier [J and I]. With this one [blind], you can adapt to it. And when you’re here, your vision can’t get worse.”

### Health state utilities

**Fig. 2:**
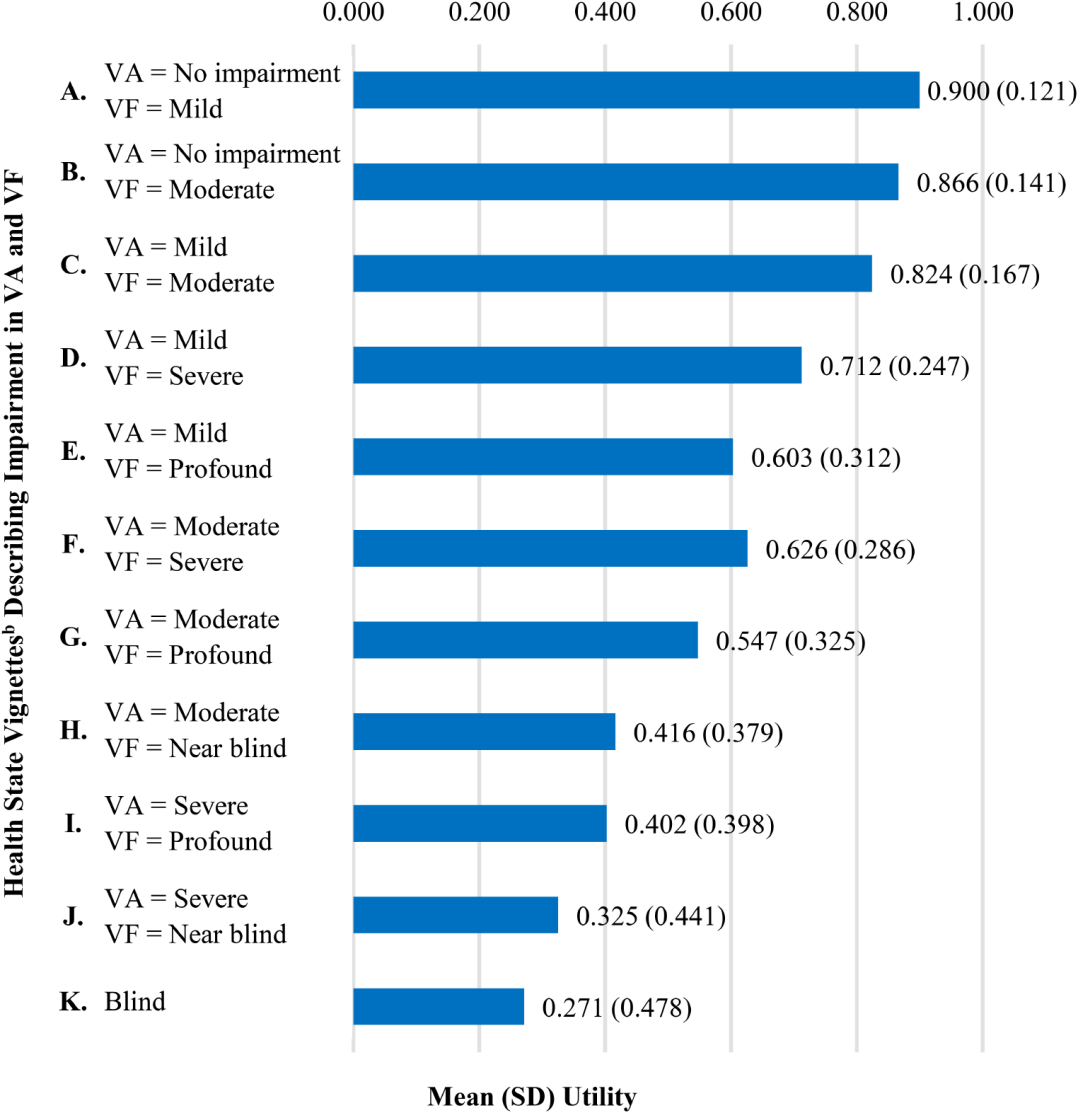
Health State Utilities^a^. Time trade-off scores are on a scale anchored with 0 representing dead and 1 representing full health. ^b^ Letters used to label health states were scrambled on participant-facing materials during interviews to ensure the labels did not correspond to health state severity. For example, health states A, B, and C were labeled with the letters Q, W, and G, respectively. *Abbreviations*: SD = standard deviation; VA = visual acuity; VF = visual field

Mean utilities decreased with greater impairment in visual acuity and visual field (Fig. 2). Health state A, with the mildest impairment, had the highest mean utility (0.900). Health state K (blind) had the lowest mean utility (0.271). This pattern of mean utilities was consistent across the three interview locations. Mean utilities for all health states were significantly different (*P* < 0.001) from the mean utility of adjacent health states (e.g., A vs. B, B vs. C…), with the exception of health states H and I, which were not significantly different from each other.

All participants rated health states A, B, and C as better than dead (utility > 0). Negative utilities were rare for health states D to G with percentages of negative scores ranging from 0.8 to 2.4%, and more common for H (6.5%), I (9.0%), and J (13.1%). Health state K (blind) had the greatest number of negative utilities (16.3%).

### Subgroup comparisons

Utilities were compared by subgroups. No differences in utility were found by age (median split, older vs. younger) or city (London, Newcastle, Edinburgh). In men and women, utilities followed the same pattern with utility decreasing as severity of impairment increased, but male participants had significantly lower utilities than female participants for all health states (Table 2). Mean utility differences between male and female participants ranged from 0.035 (*P* = 0.026) for health state A to 0.189 (*P* < 0.001) for health state I. Despite this systematic difference in absolute value of the utilities, the pattern of differences between the health state utilities was consistent among men and women. For example, utility decreases from each health state to the next most severe health state were consistent for men and women, as there was no systematic difference between genders in the difference between adjacent health states.

## Discussion

Results highlight the impact of visual impairment on quality of life and health state preference. Utilities followed expected patterns, with more severe visual impairment associated with lower mean utility. Health states with mild-to-moderate impairment had utilities ranging from 0.82 to 0.90, which are typical values for health states representing relatively mild medical conditions. The most severe health states, describing patients who are blind or near blind, had utilities ranging from 0.27 to 0.42. Utilities in this lower range are commonly reported for medical conditions associated with significant impairment and impact on quality of life, such as short bowel syndrome with daily intravenous nutrition supplementation [36] and pemphigus vulgaris [37]. The utility of 0.27 for blindness is similar to values for blindness reported in previous research [16, 38].

When generic instruments are not sensitive or feasible, utilities derived using other methods may be acceptable to HTA reviewers [26]. Due to the limitations of generic instruments in XLRP, the current study used the vignette-based method, which is the most common approach for estimating utilities associated with rare conditions [15, 37, 39–43]. Consistent with recent recommendations [23, 24], XLRP health states were based on the best available evidence, including extensive collaboration with clinicians and patients. To ensure clarity and accuracy of the vignettes, each included two photos that were systematically adjusted to represent the specific visual impairment described in each health state. Then, utilities were derived using commonly accepted TTO methods. Given the limitations of generic instruments in this population and the methodological rigor of the current study, the resulting utilities should be considered useful in economic models of treatment for XLRP.

Still, the inherent limitations of vignette-based methods should be acknowledged [23]. The utilities represent preferences for health state descriptions rather than the real-world experience of actual patients. The extent to which these utilities may differ from values derived from patients is unknown.

Another limitation is that the number of health states in a vignette-based study is determined in consideration of trade-offs between comprehensiveness and feasibility. It would be impossible to value every possible combination of visual acuity and visual field impairment. The number of health states was selected based on feasibility of the task with various numbers of health states in the pilot study. Then, the combinations of visual acuity and visual field impairment were selected to represent the health states that most commonly occur among patients in clinical settings and clinical trials. However, there are likely to be some patients with XLRP who do not fit the current health states, and when modeling these patients, utilities will need to be estimated based on values provided for health states in a similar severity range. For example, data from the current study could be used to estimate utilities for combinations of visual acuity and visual field impairment that are not represented in the current health states.

Generalizability should also be considered. The sample was recruited to match the UK’s general population with regard to key demographic variables, and interviews were conducted in three geographical regions within the UK. The pattern of results was consistent across the three locations, which adds to confidence in generalizability within the UK.

## Conclusion

In conclusion, utilities followed reasonable patterns, with lower utilities associated with more severe impairment in visual acuity and visual field. The utilities estimated in this study would be useful in CUAs assessing the value of treatments for XLRP to inform HTA decision-making about healthcare resource allocation. These utilities may also be applicable in models examining treatments for other visual conditions where reduced visual field and visual acuity are the primary symptoms. In addition, the relatively low utilities for the more severely impaired health states highlight the substantial impact of XLRP on health-related quality of life.

## Electronic supplementary material

Below is the link to the electronic supplementary material.

Supplementary Material 1

## Data Availability

Reasonable requests for study data and materials will be considered.
