# Supplementary material for: Health state utilities associated with X-linked retinitis pigmentosa (XLRP)
Source: Eur J Health Econ. 2025 Mar 17;26(7):1263–73. doi: 10.1007/s10198-025-01761-y (PMC12431885; doi:10.1007/s10198-025-01761-y)
Supplement: Supplementary file 1 — Supplementary Material 1 [file 10198_2025_1761_MOESM1_ESM.pdf]

ELECTRONIC SUPPLEMENTARY MATERIALS FOR 'HEALTH STATE UTILITIES ASSOCIATED WITH X-LINKED RETINITIS PIGMENTOSA (XLRP)'

Contents

ONLINE RESOURCE 1: LEVELS OF IMPAIRMENT IN VISUAL ACUITY AND VISUAL FIELD[a] 1

ONLINE RESOURCE 2: BACKGROUND INFORMATION PAGE 2

ONLINE RESOURCE 3: TEXT OF THE HEALTH STATE VIGNETTES 3

ONLINE RESOURCE 1: LEVELS OF IMPAIRMENT IN VISUAL ACUITY AND VISUAL FIELD[a]

Level of Impairment	Visual Acuity Range			Visual Field Range
	Decimal Notation	LogMar	US Notation	Diameter
Normal vision	1.6 – 0.8	-0.20 – 0.10	20/12 – 20/25	120°
Mild visual impairment	0.63 – 0.32	0.20 – 0.50	20/32 – 20/63	100° – 80°
Moderate visual impairment	0.25 – 0.125	0.60 – 0.90	20/80 – 20/160	60° – 40°
Severe visual impairment	0.1 – 0.05	1.00 – 1.30	20/200 – 20/400	20° – 16°
Profound visual impairment	0.04 – 0.02	1.40 – 1.70	20/500 – 20/1000	12° – 8°
Near blind	less	2.00	less	6° – 4°
Blind	0	3.00	NLP	2° – 0°

[a] This table was derived from the International Council of Ophthalmology report, "Visual Standards – Aspects and Ranges of Vision Loss with Emphasis on Population Surveys" [1]. The severity levels in this table were used as the structure for the health states in the current study.

1. International Council of Ophthalmology. Visual Standards – Aspects and Ranges of Vision Loss with Emphasis on Population Surveys. 29th International Congress of Ophthalmology; Sydney, Australia; April 2002.

ONLINE RESOURCE 2: BACKGROUND INFORMATION PAGE

[This page was reviewed with participants prior to presenting the health states.]

- You have a rare genetic eye disease that causes blindness over time.
- This disease is characterized by two main symptoms: reduced visual acuity and visual field impairment.
  - **Visual acuity** is how clear and sharp objects appear.
  - **Visual field** is how wide of an area your eye can see.
- Levels of impairment in visual acuity and visual field fall into **seven categories** of severity:
  1. No impairment
  2. Mild
  3. Moderate
  4. Severe
  5. Very severe[a]
  6. Near blind
  7. Blind
- This disease cannot be corrected with glasses.

[a] Based on the results of the pilot study, participants had difficulty understanding the term "profound," which is the term used in the severity levels from the International Council of Ophthalmology visual standards report presented in Online Resource 1. Therefore, level 5 of impairment was labeled as "very severe" instead of "profound" in all participant-facing materials during the main study.

ONLINE RESOURCE 3: TEXT OF THE HEALTH STATE VIGNETTES

Health State A

Visual Acuity: No Impairment

- You have **no** visual acuity impairment.
- You can see objects clearly and sharply.

Visual Field: Mild

- You have **mild** visual field impairment.
- The area that you are able to see is **slightly smaller** than it used to be.
- You **never** notice this in daylight.

Night Blindness

- You notice **a little** difficulty seeing in the dark.

Impact

- You are able to perform your daily activities **without difficulty**.
- You are concerned about the future. **Sometimes**, your visual condition has an impact on your emotions.
- Your visual impairment **does not affect your driving** during the day, but it is **difficult** to drive in the dark. In some locations, you would not be legally permitted to drive.

Health State B

Visual Acuity: No Impairment

- You have **no** visual acuity impairment.
- You can see objects clearly and sharply.

Visual Field: Moderate

- You have **moderate** visual field impairment.
- The area that you are able to see is **smaller** than it used to be.
- You can no longer see objects on the **far sides** of your visual field.
- You **notice this more in dark conditions**, like when you are outside at night.
- When walking, you **sometimes** trip or bump into objects, especially in dark conditions.

Night Blindness

- You notice **some difficulties** seeing in the dark.

Impact

- You **sometimes have difficulty** with daily activities.
- You are concerned about the future. **Sometimes**, your visual condition has an impact on your emotions.
- In the **dark**, you **cannot drive**. Driving would also be **difficult** during the day. In some locations, you would not be legally permitted to drive.

Health State C

Visual Acuity: Mild

- You have **mild** visual acuity impairment.
- Your vision is **somewhat blurred**.
- Objects **do not** appear perfectly clear and sharp.
- In most situations, you **can see well enough** to read and recognize faces.

Visual Field: Moderate

- You have **moderate** visual field impairment.
- The area that you are able to see is **smaller** than it used to be.
- You can no longer see objects on the **far sides** of your visual field.
- You **notice this more in dark conditions**, like when you are outside at night.
- When walking, you **sometimes** trip or bump into objects, especially in dark conditions.

Night Blindness

- You notice **some difficulties** seeing in the dark.

Impact

- You **sometimes have difficulty** with daily activities.
- You are concerned about the future. **Sometimes**, your visual condition has an impact on your emotions.
- In the **dark**, you **cannot drive**. Driving would also be **difficult** during the day. In some locations, you would not be legally permitted to drive.

Health State D

Visual Acuity: Mild

- You have **mild** visual acuity impairment.
- Your vision is **somewhat blurred**.
- Objects **do not** appear perfectly clear and sharp.

- In most situations, you can **see well enough** to read and recognize faces.

Visual Field: Severe

- You have **severe** visual field impairment.
- Your visual field is constricted. You only see objects that are in the **middle of your visual field**.
- You notice this in **all** situations.
- When walking, you **often** trip or bump into objects.

Night Blindness

- It is **very difficult** for you to see in the dark.

Impact

- You have **difficulty** with daily activities such as finding things in your home, shopping, and getting around when you leave home.
- You are concerned about the future. **Sometimes**, your visual condition has an impact on your emotions.
- You **cannot drive**.

Health State E

Visual Acuity: Mild

- You have **mild** visual acuity impairment.
- Your vision is **somewhat blurred**.
- Objects **do not** appear perfectly clear and sharp.
- In most situations, you **can see well enough** to read and recognize faces.

Visual Field: Very Severe

- You have **very severe** visual field impairment.
- You can only see **straight ahead of you**.
- You notice this in **all** situations.
- You **sometimes** need a **mobility cane (white stick)** when walking around outside the home to detect obstacles and assist with safely navigating your path.

Night Blindness

- You **cannot** see in the dark.

Impact

- You have **difficulty** with daily activities such as finding things in your home, shopping, and getting around when you leave home.

- You are concerned about the future. **Sometimes**, your visual condition has an impact on your emotions.
- You **cannot drive**.

Health State F

Visual Acuity: Moderate

- You have **moderate** visual acuity impairment.
- Your vision **is blurred**.
- To read, you **often** need magnifying glasses or other visual aids like a screen where the image can be enlarged.
- It is **sometimes** difficult to recognize faces.

Visual Field: Severe

- You have **severe** visual field impairment.
- Your visual field is constricted. You only see objects that are in the **middle of your visual field**.
- You notice this in **all** situations.
- When walking, you **often** trip or bump into objects.

Night Blindness

- It is **very difficult** for you to see in the dark.

Impact

- You have **difficulty** with daily activities such as finding things in your home, shopping, and getting around when you leave home.
- You are concerned about the future. **Sometimes**, your visual condition has an impact on your emotions.
- You **cannot drive**.

Health State G

Visual Acuity: Moderate

- You have **moderate** visual acuity impairment.
- Your vision **is blurred**.
- To read, you **often** need magnifying glasses or other visual aids like a screen where the image can be enlarged.
- It is **sometimes** difficult to recognize faces.

Visual Field: Very Severe

- You have very severe visual field impairment.
- You can only see **straight** ahead of you.
- You notice this in **all** situations.
- You **sometimes** need a **mobility cane (white stick)** when walking around outside the home to detect obstacles and assist with safely navigating your path.

Night Blindness

- You **cannot** see in the dark.

Impact

- You have **difficulty** with daily activities such as finding things in your home, shopping, and getting around when you leave home.
- You are concerned about the future. **Sometimes**, your visual condition has an impact on your emotions.
- You **cannot drive**.

Health State H

Visual Acuity: Moderate

- You have **moderate** visual acuity impairment.
- Your vision **is blurred**.
- To read, you **often** need magnifying glasses or other visual aids like a screen where the image can be enlarged.
- It is **sometimes** difficult to recognize faces.

Visual Field: Near Blind

- You are **nearly blind**.
- You can only see **straight ahead of you**, as if you are in a **tunnel**.
- You notice this in **all** situations.
- You **always** need a **mobility cane (white stick)** when walking around outside the home to detect obstacles and assist with safely navigating your path.

Night Blindness

- You **cannot** see in the dark.

Impact

- You **need assistance** with many daily activities such as finding things in your home, shopping, and getting around when you leave home.

- You are concerned about the future. **Sometimes**, your visual condition has an impact on your emotions.
- You **cannot drive**.

Health State I

Visual Acuity: Severe

- You have **severe** visual acuity impairment.
- Your vision is **very blurred**.
- To read, you **always** need magnifying glasses or other visual aids like a screen where the image can be enlarged.
- You **often cannot** recognize faces.

Visual Field: Very Severe

- You have **very severe** visual field impairment.
- You can only see **straight ahead of you**.
- You notice this in **all** situations.
- You **sometimes** need a **mobility cane (white stick)** when walking around outside the home to detect obstacles and assist with safely navigating your path.

Night Blindness

- You **cannot** see in the dark.

Impact

- You **need assistance** with many daily activities such as finding things in your home, shopping, and getting around when you leave home.
- You are concerned about the future. **Sometimes**, your visual condition has an impact on your emotions.
- You **cannot drive**.

Health State J

Visual Acuity: Severe

- You have **severe** visual acuity impairment.
- Your vision is **very blurred**.
- To read, you **always** need magnifying glasses or other visual aids like a screen where the image can be enlarged.
- You **often cannot** recognize faces.

Visual Field: Near Blind

- You are **nearly blind**.
- You can only see **straight ahead of you**, as if you are in a **tunnel**.
- You notice this in **all** situations.
- You **always** need a **mobility cane (white stick)** when walking around outside the home to detect obstacles and assist with safely navigating your path.

Night Blindness

- You **cannot** see in the dark.

Impact

- You **need assistance** with many daily activities such as finding things in your home, choosing clothes to wear, shopping, and getting around when you leave home.
- You are concerned about the future. **Sometimes**, your visual condition has an impact on your emotions.
- You **cannot drive**.

Health State K

Visual Acuity: Blind

- You are **blind**.

Visual Field: Blind

- You are **blind**.

Night Blindness

- You **cannot** see in the dark.

Impact

- You **need assistance** with many daily activities such as finding things in your home, choosing clothes to wear, shopping, and getting around when you leave home.
- You are concerned about the future. **Sometimes**, your visual condition has an impact on your emotions.
- You **cannot drive**.
